# Continuous Electrical Current and Zinc Sulphate Administered by Transdermal Iontophoresis Improves Skin Healing in Diabetic Rats Induced by Alloxan: Morphological and Ultrastructural Analysis

**DOI:** 10.1155/2014/980232

**Published:** 2014-08-28

**Authors:** Lucas Langoni Cassettari, Pedro Colli Rocha Dias, Amanda Natália Lucchesi, Maurício Ferraz de Arruda, Érika Veruska Paiva Ortolan, Mariângela Esther A. Marques, César Tadeu Spadella

**Affiliations:** ^1^Faculty of Medicine, São Paulo State University (UNESP), 18618-970 Botucatu, SP, Brazil; ^2^Graduate Program in General Basis of Surgery, Faculty of Medicine, São Paulo State University (UNESP), 18618-970 Botucatu, SP, Brazil; ^3^Municipal Institute of Higher Education, IMES-FAFICA, 55030-902 Catanduva, SP, Brazil; ^4^Department of Surgery and Orthopedics, Faculty of Medicine, São Paulo State University (UNESP), 18618-970 Botucatu, SP, Brazil; ^5^Department of Pathology, Faculty of Medicine, São Paulo State University (UNESP), 18618-970 Botucatu, SP, Brazil

## Abstract

*Purpose.* Evaluated the effects of continuous electrical current (CEC) or zinc administrated by transdermal iontophoresis (Zn+TDI).* Methods.* 120 male Wistar rats were submitted to an incision surgery at the anterior region of abdomen and distributed into 6 experimental groups with 40 animals: 3 diabetic groups and 3 normal groups, untreated and treated with CEC alone or with Zn + TDI. Each group was further divided into 4 subgroups with 10 rats each to be evaluated on the 4th, 7th, 14th, and 21st day after surgery. In each period, clinical and laboratory parameters from the animals were analyzed.* Results*. The analysis by optical and scanning electron microscopy showed a delay in the phases of wound healing in diabetic rats without treatment in all periods of the experiment; breaking strength (BS) was significantly reduced in skin scars of untreated diabetic rats when compared to other groups. In contrast, BS in skin scars of nondiabetic groups and diabetic rats treated with Zn + TDI showed significant increase in those, besides not presenting delayed healing.* Conclusion*. Electrical stimulation of surgical wounds used alone or in association with zinc by TDI is able to consistently improve the morphological and ultrastructural changes observed in the healing of diabetic animals.

## 1. Introduction

Studies in humans and animals show that DM slows contraction and closure of the incision, decreases the breaking strength of incisions and anastomosis, and acts negatively on all phases of wound healing [1–5].

Long-term research has shown that effective control of hyperglycemia plays an important role in the control of chronic diabetic lesions and may, therefore, improve the changes of the healing process of surgery wounds [6, 7].

Additionally, currently, the search for an effective treatment to improve wound healing in diabetic patients remains a challenge for researchers. There are numerous therapies tested in clinical and experimental studies in order to improve wound healing in normal and diabetic hosts. Among them, we highlight the oral, parenteral, or topical substances derived from herbal extracts administration, insulin, zinc, chromium, laser irradiation, extracorporeal shock wave pathway, growth factors, artificial skin, culture of epithelium, with or without dermal components, and electrical stimulation of tissues, isolated or associated with administration of soluble ions by transdermal iontophoresis (TDI) [8–14].

Among the various substances used by TDI, zinc has particular interest in this study because it is an inherent part of the insulin acts in carbohydrates metabolism and collagen and is intrinsically related to the tissue repair process [15]. Postoperatively, zinc levels may be reduced in diabetic men and animals. This deficiency can lead to decreased formation of connective tissue repair and alteration of wound healing process [16].

In current literature, there are few clinical and experimental studies on wound healing in diabetics using CEC or Zn + TDI [17–19].

A recent study in our laboratory demonstrated that hyperglycemic diabetic rats showed a significant reduction of breaking strength of skin scarring, especially in the most vulnerable stages of incisions as dehiscence and infection. However, this change was completely reversed by the topical treatment of incisions TDI + Zn in the early stages of the healing process. Moreover, despite the low values of breaking strength found in the scars of diabetic animals was observed that the amount of hydroxyproline (P-OH) present in the tissue not differ from the values found in normal or diabetic rats treated with Zn + TDI in all phases of the healing process. This finding suggested the hypothesis that the decrease of breaking strength in surgical scars skin of diabetic animals may be more related to morphological changes present in the early stages of tissue repair, and later, the level of organization and maturation of collagen deposited the scars, than the amount of this protein in the tissues.

The objective of this study was to evaluate the effects of CEC and TDI + Zn on morphological and ultrastructural changes of the healing process of the skin of alloxan-induced diabetic rats and to establish the correlation between low values of breaking strength found incisions in these animals with histopathological changes observed. We hope that our findings can clarify the issues reported here and also contribute to a better therapeutic approach to changes caused by the DM in the process of healing of surgical wounds.

## 2. Methods

### 2.1. Animals and Diabetes Induction

Use of laboratory animals was approved by the Animal Experimentation Ethics Committee of our institution. One hundred and twenty male Wistar rats, with approximately 250 g, from the Central Animal Laboratory, Faculty of Medicine, UNESP, Botucatu-SP, were used in this experiment.

Animals were randomly assigned into 6 experimental groups, with 20 animals each, as follows: NG/NT is nondiabetic control group consisting of normal mice without any treatment of abdominal surgical scar; DG/NT is diabetic group consisting of diabetic hyperglycemic rats without any treatment of abdominal surgical scar; NG/CEC is nondiabetic group, treated with continuous electrical current; DG/CEC is diabetic hyperglycemic group trated with CEC; NG/Zn + TDI is nondiabetic group, treated with zinc sulfate, administered by transdermal iontophoresis; DG/Zn + TDI is diabetic hyperglycemic group, treated with zinc sulfate and administered by Zn + TDI. Each experimental group was further divided into 4 subgroups with 10 animals each, to be evaluated and sacrificed, respectively, in four different times of the experiment, on the 4th, 7th, 14th, and 21st day after surgery.

Diabetes was induced by alloxan administered intravenously at a dose of 42 mg/kg of body weight using one of the tail veins. We only used animals with clinical signs of severe diabetes and fasting glucose ≥250 mg/dL in two successive determinations (seven and 14 days after diabetes induction).

### 2.2. Analyzed Parameters

At the beginning of the experiment (14th day of follow-up or diabetes onset) and at each time point of follow-up, clinical (body weight, water intake, food intake, and diuresis) and laboratory (blood glucose, urinary glucose, glycosylated hemoglobin, and plasma insulin) parameters were analyzed. Clinical parameters were obtained using individual metabolic cages. Blood glucose was determined by standard enzymatic method (Johnson & Johnson's), glycosylated hemoglobin by agarose gel electrophoresis (Sebia, France) and insulin by radioimmunoassay (Diagnostic Products Corporation, USA). At sacrifice, a segment of the skin containing the incision was removed from each rat to histopathological analysis by light microscopy, ultrastructural analysis by scanning electron microscopy and measure breaking strength (BS) of the scars. After performing the biomechanical assays representative fragments of the scars were also obtained for dosage of tissue OH-P using the Switzer method.

### 2.3. Technical Procedures

All invasive procedures on animals were performed under general anesthesia using ketamine (100 mg/kg of body weight) + xylazine (50 mg/kg of body weight), administered intramuscularly (Rhobifarma Ind. Farmacêutica Ltda, Hortolândia-SP). Blood samples at sacrifice were obtained by cardiac puncture, with chest open. Euthanasia was by exsanguination, followed by the section of the vena cava infradiaphragmatic.

The surgical wounds were performed on the 15th day of normal follow-up or of diabetes induction with the animals under 12-hour fasting. After general anesthesia and trichotomy of the anterior region of the abdomen, the rats were placed on a flat surface with their limbs extended, and antisepsis of the abdomen was performed by 2% iodinated. A median, medium-umbilical incision with 4 cm in length was performed with interest in the epidermis and dermis. The incision was immediately closed with 4-0 nylon strands (Ethicon Inc., USA) using simple separate stitches at a distance of 8 mm from each other.

To treat the surgical wounds with Zn + TDI, two electrodes were positioned on the corneal layer of the epidermis of each animal. The first was placed near the lower angle of the abdominal incision and the second in the interscapular dorsal region. Both electrodes were connected to a continuous-current electrostimulator (Ibramed, São Paulo, Brazil). The abdominal electrode was connected to the anode (positive) of the device and that located on the dorsal region to the cathode (negative). The electric current emitted standardized amplitude of 2 mA, with 10-minute treatment sessions. Each session was performed at four different times in the postoperative period, of which the first was immediately after the performance of the surgical incision (immediate PO) and the other on the three subsequent days (1st, 2nd, and 3rd PO).

**Figure 1:**
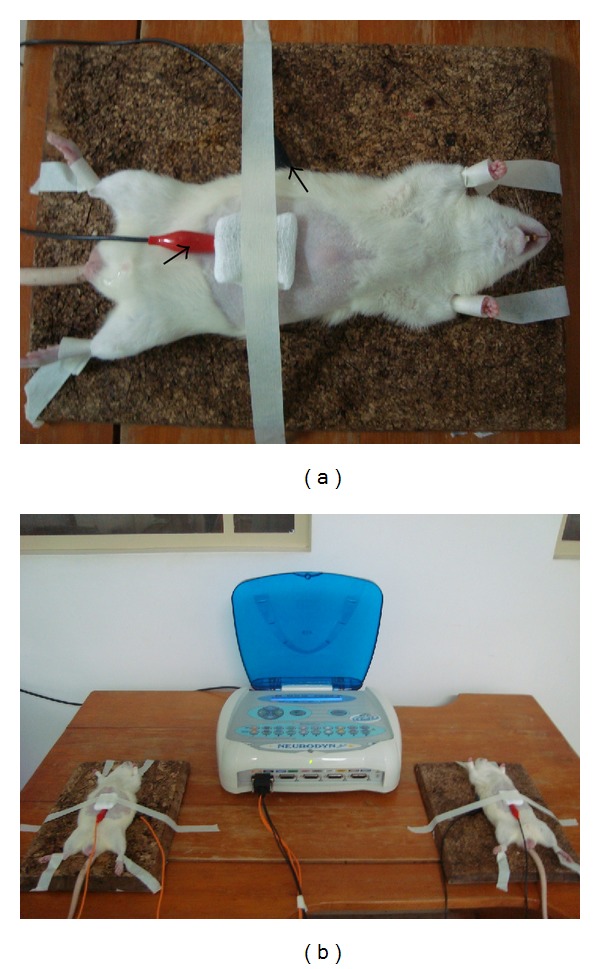
Illustration of the preparation of animals for treatment by means of iontophoresis: (a) detail of the animal with electrodes fixed in the anterior region of the abdomen and on the dorsal region (arrows) for treatment commencement; (b) animals during the electrotherapeutic treatment.

According to the proposed treatment, a small gauze pad moistened with 4 mL of zinc sulphate solubilized at the concentration of 100 mg/kg of body weight was interposed between the electrode positioned on the base of the abdominal incision and the corneal layer of the epidermis for each experimental group, as shown in Figure 1.

### 2.4. Statistical Analysis

The study of the clinical, laboratory, and biomechanical variables, according to the 6 experimental groups and the 4 sacrifice times, was conducted by means of 6 × 4 factorial analysis of variance, in a completely randomized design, complemented by the Tukey's multiple comparisons test for homogeneous or parametric variables or by Mann-Whitney and Kruskal-Wallis' nonparametric analysis for the variables showing results with a heterogeneous distribution. All the statistical discussions in the study were performed at the level of significance of 5% or *P* < 0.05.

## 3. Results

### 3.1. Clinical and Laboratory Findings

**Figure 2:**
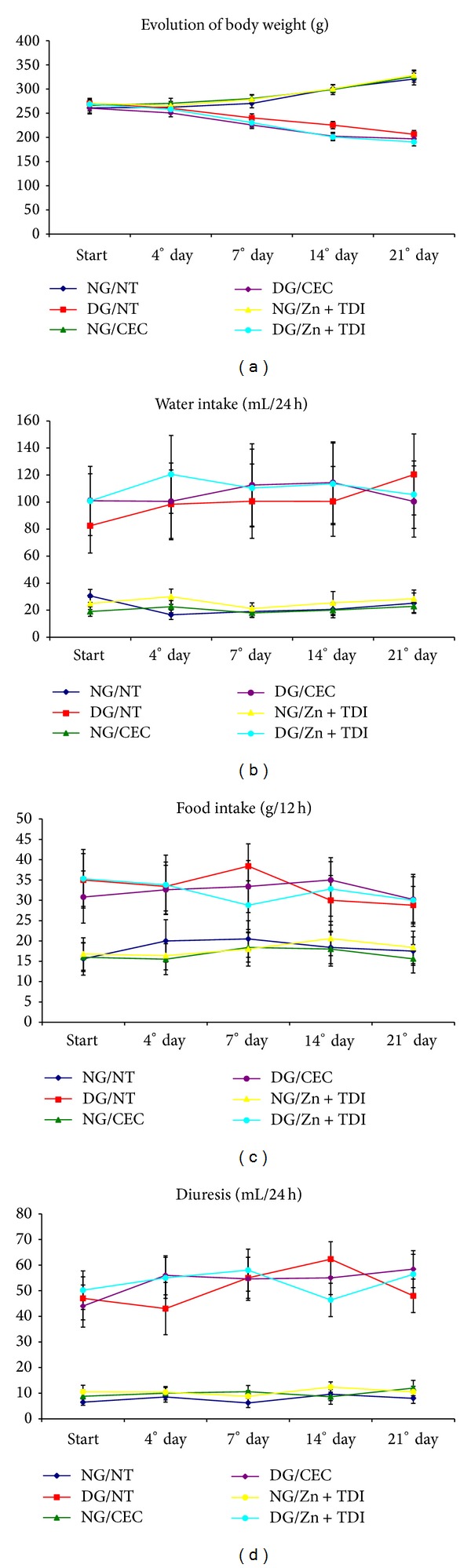
Graphs showing the variation of clinical parameters in six experimental groups during follow-up: (a) body weight (g); (b) water intake (mL/24 h); (c) food intake (g/12 h); (d) diuresis (mL/24 h).

**Figure 3:**
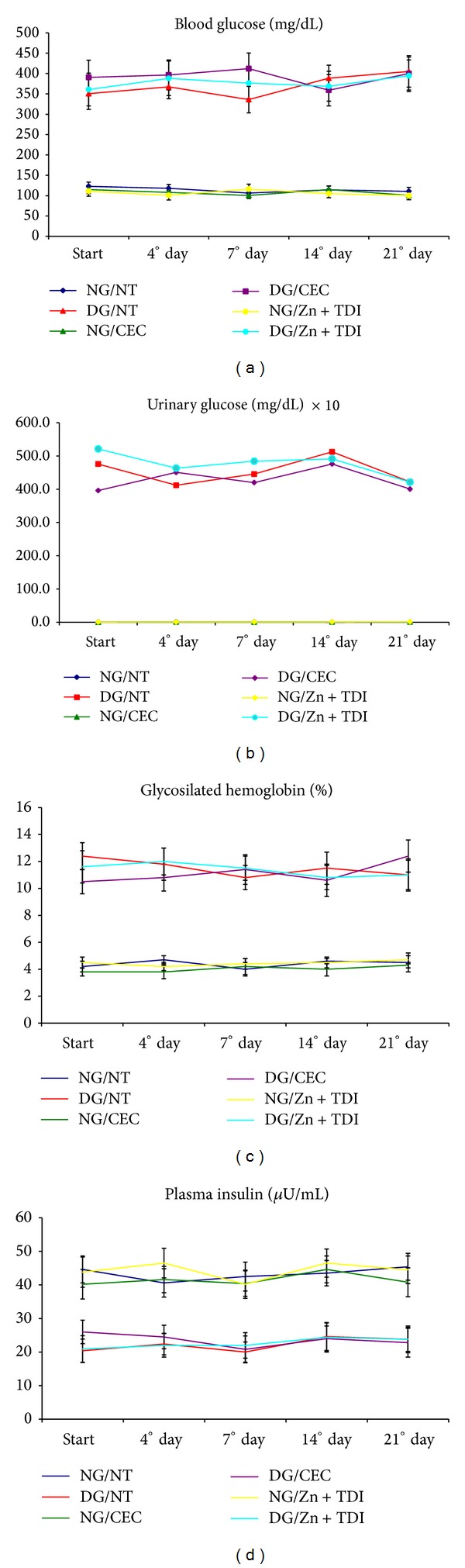
Graphs showing the variation of laboratory parameters in six experimental groups during follow-up: (a) blood glucose (mg/dL); (b) urinary glucose (mg/dL); (c) glycosylated hemoglobin (%); (d) plasma insulin (*μ*IU/mL).

Nondiabetic rats showed clinical and laboratory parameters compatible with those observed in normal animals of the same strain in all evaluation periods of the experiment. In contrast, diabetic rats without any treatment of hyperglycemia progressed to severe loss of body weight and significant increase in water intake, food intake, and urine output when compared with animals normal nondiabetic subjects (*P* < 0.001). The blood glucose levels, urine glucose, and glycosylated hemoglobin were consistently elevated in the diabetic mice groups, with plasma insulin values being significantly lower (Figures 2 and 3).

### 3.2. Morphological Findings by Light Microscopy

#### 3.2.1. Contraction of the Wound and Reepithelialization of Epithelial Surface

Surgical wounds DG/NT rats had completely removed and no edges and reepithelialization in the 4th and 7th. Moderate contraction of the wound was observed in animals DG/CEC on the 4th postoperative day with reepithelialization of the epithelial surface still absent in this period of review, but almost complete on postoperative day 7. Moreover, both the contraction of the wound and the reepithelialization was complete in rats DG/TDI + Zn, on the 4th postoperative day.

**Figure 4:**
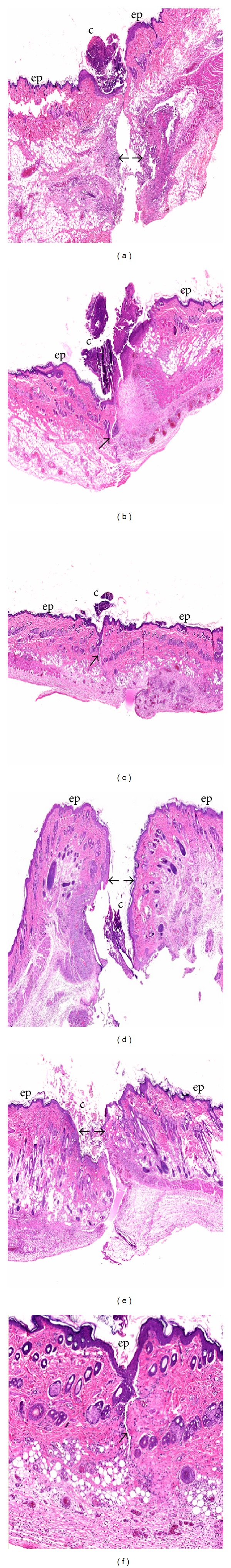
Morphological findings observed in normal rat, nondiabetic ((a), (b), and (c)) and hyperglycemic diabetic ((d), (e), and (f)), untreated or incisions treated with CEC and Zn + TDI, on the 4th postoperative day, respectively. Note the fibrin crust leukocyte (c) and the almost complete contraction of surgical margins (arrows) and reepithelialization of advanced epithelial surface (ep) in diabetic animals treated with Zn + TDI compared to nondiabetic rats treated with incisions or treated with CEC alone (H & E 20x).

These processes have been accelerated more under normal nondiabetic mice (NG), especially in the groups treated with CEC and Zn + TDI. In the latter group, the epithelial edges were already almost completely epithelized on the 4th postoperative day (Figure 4).

#### 3.2.2. Inflammatory Process and Proliferation of Fibroblasts and Vascular Endothelial Cells

On the 4th postoperative day, surgical wounds in rats DG/NT showed intense inflammatory infiltrate, predominantly composed of neutrophils, and this process is continued until the 7th postoperative day, with the same characteristics. In this period, little proliferation of fibroblasts and vascular endothelial cells was observed. In contrast, in the 4th PO, surgical wounds in rats of NG/NT or NG or DG treated with CEC or Zn + TDI, showed inflammatory infiltrate of moderate intensity, predominantly consisting of macrophages, which was replaced on postoperative day 7, for tissue granulation composed of fibroblasts, vascular endothelial proliferation and collagen deposition. Morphological inflammatory process in diabetic rats treated with CEC or Zn + TDI did not differ in the light microscopy, and was consistent with those found in rats treated or untreated NG in normal rats untreated, or treated with CEC or Zn + TDI.

#### 3.2.3. Deposition and Organization of Collagen Fibers

Fibroblast proliferation and collagen fiber formation was scarce in animals DG/NT until the 7th postoperative day. Deposition of dense collagen, with disorganized arrangement of fibers below the epithelial surface, was observed in this group only from the 14th postoperative day. In contrast, mice NG or DG treated with CEC or Zn + TDI showed progressive deposition of collagen fibers in the scar as early as the 4th postoperative day. Dense collagen arranged horizontally below the epithelial surface was observed in these groups from the 7th postoperative day. Qualitative and organizational differences in morphological structure of collagen deposition in the scar of diabetic rats, whose incisions were treated with CEC and Zn + TDI, when compared with mice where the incisions were not treated were evident.

### 3.3. Ultrastructural Findings by Scanning Electron Microscopy

**Figure 5:**
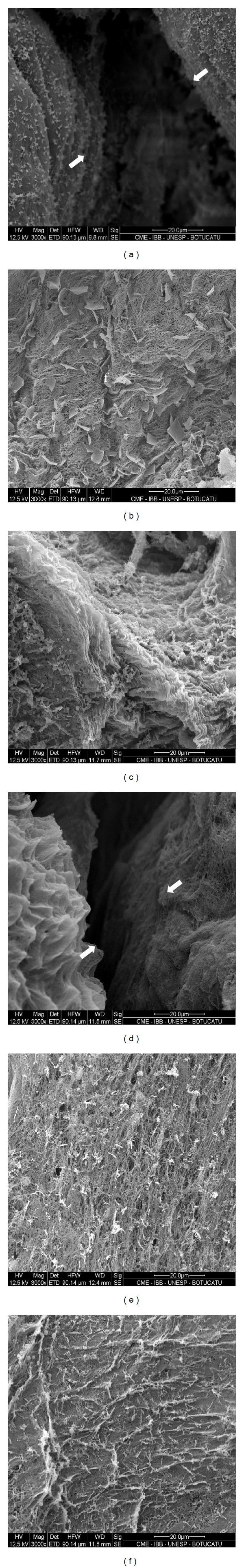
Photomicrographs of surgical scars from the skin of normal rat ((a), (b), and (c)) and diabetic ((d), (e), and (f)), sacrificed on postoperative day 7. Note that the process of reepithelialization of incisional surgery wounds is still incomplete (arrows) in rats of NG/NT and DG/NT, the largest increase ((a) and (d)). Note the disorganized collagen deposition thin, with multiple fenestrations (f) in NG and DG rats with incisions treated with CEC alone ((b) and (e)) compared with the deposition of dense collagen with few fenestrations observed in mouse NG and NG with incisions treated with Zn + TDI ((c) and (f)) (ME - 3000x).

On postoperative day 7 it was observed that collagen deposited in scars treated with CEC alone in rats of groups NG and DG showed characteristically thin and disorganized pattern although presenting multiple fenestrations between the collagen fibers, in contrast to the deposition of collagen dense, with few fenestrations observed on postoperative day 7 of scars treated with Zn + TDI, both in mice NG, as the DG. It was observed, however, that the deposition of collagen fibers was more pronounced in normal animals, when compared to diabetics (Figure 5).

**Figure 6:**
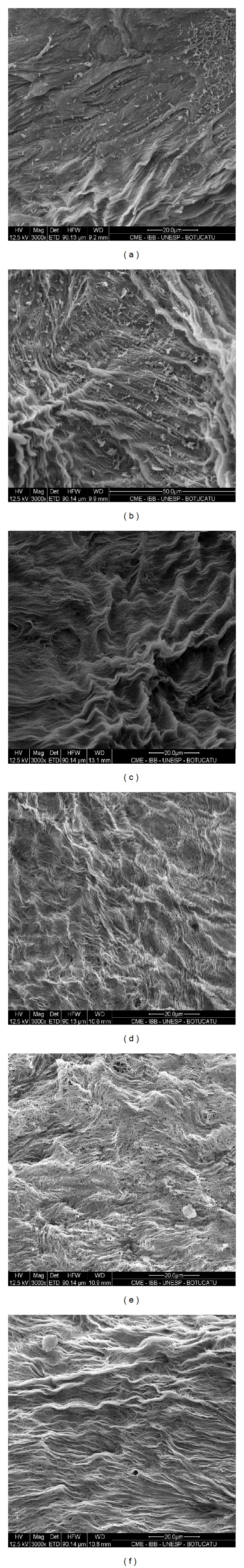
Photomicrographs of surgical scars from the skin of normal rat ((a), (b), and (c)) and diabetic ((d), (e), and (f)), sacrificed on postoperative day 21, showing organized deposition of dense, wavy provisions collagen horizontally on the epithelial surface, surgical wounds in rats of NG/NT (a), NG/CEC (b), NG/Zn + TDI (c), and DG/Zn + TDI (f). Note that this ultrastructural pattern of collagen deposition was not observed in scars of untreated (d) or treated with CEC alone (e) (EM - 3000x) diabetic rats.

On the 14th postoperative day, it was observed that healthy animals with untreated or treated incisions with CEC alone or TDI + Zn showed collagen deposition clearly dense, thicker, wavy and evenly distributed along the epithelial surface, when compared with animals DG/NT or DG/CEC, where there was the deposition of thinner, disorganized collagen fibers, yet having fenestrations between them. Moreover, in mice DG/Zn + TDI we observed an organizational pattern of the collagen fibers exceeding that observed in DG rats untreated or treated with CEC alone. 21-day follow-up, the photomicrographs showed deposition of dense, wavy collagen, neatly arranged in the horizontal direction, both in normal mice scars with untreated or treated incisions with CEC alone or Zn + TDI, as in diabetic rats whose incisions were treated with Zn + TDI. This finding, however, was not observed in mice DG/NT or treated with CEC alone, where also noted the predominance of collagen thinner, disorganized fibers deposited (Figure 6).

### 3.4. Correlation between the Breaking Strength and the Morphological and Ultrastructural Findings

**Figure 7:**
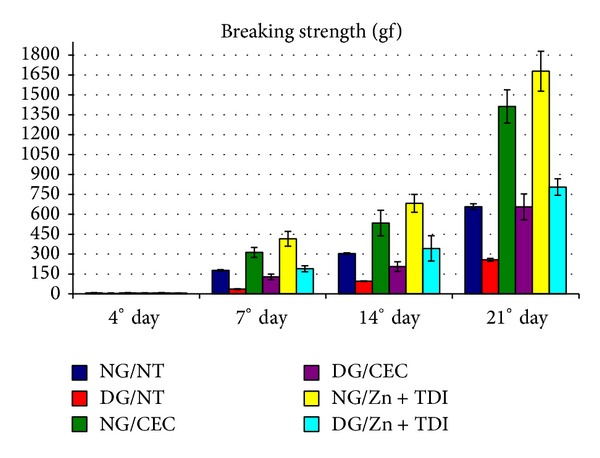
Variation in breaking strength values (gf) of scars produced in the skin of rats in the 6 experimental groups sacrificed at the 4 experimental moments.

Figure 7 shows the mean values of breaking strength (BS) found in surgical scars of animals in this experiment, obtained in a previous study conducted in our laboratory [20]. We observe that the BS of the scars was extremely low in animals DG/NT at all times of the experiment compared with normal untreated animals or normal and diabetic patients undergoing treatments with CEC alone or Zn + TDI. However, this finding was reversed in diabetic animals when used CEC alone or Zn + TDI, with values, more expressive in animals DG/Zn + TDI.

## 4. Discussion

Numerous therapeutic adjuvants have been proposed in order to modify the abnormal response of the diabetic organism during the healing of surgical wounds. In this study, we demonstrated that hyperglycemic diabetic rats without any treatment of the incision showed significant morphological changes in the healing process of the wound characterized by contraction and epithelialization of epithelial edges, increasing the intensity and prolongation of neutrophil-mediated inflammatory phase delay and as macrophage migration, proliferation of fibroblasts and vascular endothelial cells and delay of the production of collagen, when compared to normal nondiabetic untreated rats or normal or diabetic rats which surgical incisions were treated with CEC, or Zn + TDI.

Komesu et al. (2004) [21] studied the morphological effects of diabetes on the healing process of the skin excised from alloxan-induced diabetic rats; they observed that the initial phase of inflammation mediated by neutrophils, by neutrophlis, started more slowly, and continued after the 3rd postoperative day. During this period, these cells should already have left the injured area, to be replaced by macrophages and subsequently by fibroblasts. Low density neutrophils were also observed by these authors, until the 3rd day of healing, however, with a significant increase in the number of these cells after 7 days of surgery.

Researches by various authors show that the quality of the inflammatory response of tissues to injury is an important stage on the wound healing process [22–24]. Thus, defects observed in the inflammatory phase of wound healing in diabetic patients can reduce the expression of growth factors, with subsequent failure in the processes of fibroblast proliferation, angiogenesis and synthesis of collagen [25–27].

Our results also showed a marked impairment in the ability of contraction and reepithelialization of skin edges of the wound in untreated diabetic rats compared with control normal rats untreated or normal and diabetic rats treated with incisions or CEC or Zn + TDI.

Macrophages play a crucial role in the transition between the inflammatory phase and the phase of fibroblast proliferation and collagen synthesis, necessary for tissue repair. These processes, however, are altered in diabetic patients, characteristically delayed relative to normal, as observed in this study.

It is known that the formation of the initial epithelial barrier depends on the contraction of mesenchymal cells, which bring the edges of the wound closer to reduce the size of the injured area. Cells of the epidermis and dermis start to move across the wound, and aided by cell proliferation, epithelial surface ends up by reset, first on the edge of the residual dermis and then woven about the newly formed granulation, which is gradually filled by a new extracellular matrix produced by fibroblasts, which is being progressively replaced by collagen to fill the entire space below the dermis reviled [28, 29].

This study showed, however, that the process of deposition and organization of collagen in the skin scars is also defective in hyperglycemic nontreated diabetic rats, consisting of loose and low density collagen fibers, even on the 14th postoperative day when compared with respective treated or nontreated controls. Analyzing by scanning electron microscopy, it was found that unlike control animals, the collagen deposition in diabetic animals is markedly disorganized and composed of immature fibers with numerous “empty” not yet filled in the injured area. This finding has already been observed in our laboratory in anastomosis of small and large intestine of diabetic rats without any treatment [30]. Both studies, however, revealed that there was no change in the content of collagen in skin scars and intestines being found normal levels of OH-P and tissue protein in these tissues.

The results of scanning electron microscopy are very illustrative with regard to improving the quality of healing in relation to the proposed treatment.

According to Parizotto (1998) [31], during the first week after injury, the edges of the wounds are still not completely epithelialized, a fact corroborated in our study on postoperative day 7 in normal and diabetic animals without treatment. However in mice treated with CEC alone or Zn + TDI in the same period, complete epithelialization of surgical margins was observed, indicating the effectiveness of the method in the early stages of healing. The presence of ripples on collagen fibers is featuring acceleration in the formation and maturation of the same, with this fact being related to increased mechanical strength of wound healing [32]. The presence of hair follicles holes emerging in epidermis correlates with a high degree of healing [33]. These aforementioned factors were also observed in our study, from the 14th postoperative day in normal and diabetic animals treated with Zn + TDI.

These results suggest that the decrease in breaking strength (BS), observed in scarring of the skin of animals in this study is much more related to prolongation of the inflammatory phase and delay the onset of fibroplasia (in the early stages of healing) and the degree organization and maturation of collagen deposition in the scar (in later stages) than the amount of this protein in tissues.

Onodera et al. (2004) [34], however, trying to establish a correlation between collagen synthesis with the BS of anastomosis in the colon of diabetic rats, concluded that the decrease in strength in these anastomosis was much more dependent on the collagen newly formed on the scar, than the total content of collagen present in the tissue.

Recent study by our research group showed that practiced surgical incisions in the abdominal wall of diabetic rats, 3 months after induction of diabetes, showed significant decrease of BS, difficulty to promote wound contraction and increase collagen density in scars, especially in the early stages of healing.

In this study, we show that the derived scarring of the DM can be completely reversed by treatment of the incisions with electrical stimulation of tissues, applied alone or in combination with zinc sulfate by TDI. Both treatments were effective in modifying the changes observed in diabetic, nontreated animals, both in the inflammatory phase of healing, as the phases of cell proliferation, synthesis, organization and remodeling of the collagen.

However, the benefits were more evident with the use of the Zn + TDI in all periods of evaluation conducted. Beneficial effects of TDI and its variants (electroporation, phonophoresis, and chemical enhancers systems) have also been observed in a wide variety of situations and clinic conditions [14, 35, 36]. However, there are few publications on the use of these methods in order to correct the disorders of surgical wound healing practiced in the skin of diabetic hosts, especially using substances with positive effects on hyperglycemia and/or the healing process, as insulin, growth factors, chromium, and zinc.

Agren (1990) [37], using TDI by electroporation associated with the administration of transforming growth factor beta 1 (TGF beta-1), diabetic (db/db) factor, observed healing rate was higher than in untreated groups, therefore 5–7 days after intradermal application of the substance, the beds of the wounds showed a significant increase in the rate of epithelialization, collagen synthesis and angiogenesis.

Positive effects on surgical wound healing in normal and diabetic animals and men have also been reported with the use of zinc given orally, enteral, intraperitoneal or topic. However, the results of these searches are limited or controversial, given that the benefits of therapy have only been well documented in individuals with proven this trace element in disability on organism [16, 38].

On the other hand, a study conducted by Cornwall (1981) [39] showed that patients underwent bilateral amputation of the legs and who developed ulcerations on the stumps had shrinkage and faster closing of the ulcerated areas, when treated with Zn + TDI, than those belonging to historical control service, where treatment was not employed group.

Esen et al. (2006) [40] also observed that administration of zinc associated with TDI promoted local dilation of blood vessels of the forearm in normal subjects, resulting in analysis by Doppler, better fluidity of blood. The authors suggested that this property could promote wound healing in diabetic subjects due to the mineral beneficial effect on the aggregation of blood and platelets.

Previous studies in our laboratory [20, 41] have already proven that Zn + TDI significantly increased the tensile strength of skin incisions practiced in the abdomen of the animals in this study, with no change in the levels of collagen present in these scars, measured by the concentration of OH-P tissue.

This study, however, demonstrated that treatment of incisions with Zn + TDI completely changed the course of morphological and ultrastructural changes observed in the healing process of the skin of diabetic hyperglycemic animals without any treatment of the incision. Similar results were observed with the use of electrical stimulation (ES) on tissues; however, the administration of zinc sulfate by TDI was more effective in acute phase, as in the later stages of the process.

## 5. Conclusion

The electrical stimulation (ES) to surgical wounds, used alone or in combination with the administration of zinc sulfate by TDI, is able to consistently improve the morphological and ultrastructural changes seen in the healing of diabetic animals process, even when the hyperglycemic state is maintained. The benefits of ES, however, were more evident when it was used in combination with the zinc sulfate. Our results suggest that the decrease of breaking strength of incisions in diabetic animals is much more associated with morphological and ultrastructural alterations present in the scars, than the content of collagen deposited in them.

## References

[1] Goodson WH, Hunt TK (1979). Wound healing and the diabetic patient. *Surgery Gynecology and Obstetrics*.

[2] Spanheimer RG, Umpierrez GE, Stumpf V (1988). Decreased collagen production in diabetic rats. *Diabetes*.

[3] Tellechea A, Kafanas A, Leal EC (2013). Increased skin inflammation and blood vessel density in human and experimental diabetes. *International Journal of Lower Extremity Wounds*.

[4] Khanna S, Biswas S, Shang Y (2010). Macrophage dysfunction impairs resolution of inflammation in the wounds of diabetic mice. *PLoS ONE*.

[5] Minossi JG, Lima FO, Caramori CA (2014). Alloxan diabetes alters the tensile strength, morphological and morphometric parameters of abdominal wall healing in rats. *Acta Cirurgica Brasileira*.

[6] Nathan DM, Bayless M, Cleary P (2013). Diabetes control and complications trial/epidemiology of diabetes interventions and complications study at 30 years: advances and contributions. *Diabetes*.

[7] McMurry JF (1984). Wound healing with diabetes mellitus. Better glucose control for better wound healing in diabetes. *Surgical Clinics of North America*.

[8] Rabelo SB, Villaverde AB, Nicolau RA, Castillo Salgado MA, Melo MDS, Pacheco MTT (2006). Comparison between wound healing in induced diabetic and nondiabetic rats after low-level laser therapy. *Photomedicine and Laser Surgery*.

[9] Yang G, Luo C, Yan X, Cheng L, Chai Y (2011). Extracorporeal shock wave treatment improves incisional wound healing in diabetic rats. *The Tohoku Journal of Experimental Medicine*.

[10] Greenhalgh DG, Sprugel KH, Murray MJ, Ross R (1990). PDGF and FGF stimulate wound healing in the genetically diabetic mouse. *The American Journal of Pathology*.

[11] Inpanya P, Faikrua A, Ounaroon A, Sittichokechaiwut A, Viyoch J (2012). Effects of the blended fibroin/aloe gel film on wound healing in streptozotocin-induced diabetic rats. *Biomedical Materials*.

[12] Velander P, Theopold C, Bleiziffer O (2009). Cell suspensions of autologous keratinocytes or autologous fibroblasts accelerate the healing full thickness skin wounds in a diabetic wound healing. *Journal of Surgical Research*.

[13] Kloth LC (2005). Electrical stimulation for wound healing: a review of evidence from in vitro studies, animal experiments, and clinical trials. *International Journal of Lower Extremity Wounds*.

[14] Dixit N, Bali V, Baboota S, Ahuja A, Ali J (2007). Iontophoresis—an approach for controlled drug delivery: a review. *Current Drug Delivery*.

[15] Landsdown ABG (1996). Zinc in the healing wound. *The Lancet*.

[16] Moraes SP, Chaves FRB, Banci S, Rover PA, Georgetti F, Reis-Neto JA (2000). The effect of zinc and chromium on wound healing in normal and diabetic rats. *Revista do Colégio Brasileiro de Cirurgiões*.

[17] Smith J, Romansky N, Vomero J, Davis RH (1984). The effect of electrical stimulation on wound healing in diabetic mice. *Journal of the American Podiatry Association*.

[18] Lee PY, Chesnoy S, Huang L (2004). Electroporatic delivery of TGF-beta1 gene works synergistically with electric therapy to enhance diabetic wound healing in db/db mice. *Journal of Investigative Dermatology*.

[19] Thawer HA, Houghton PE (2001). Effects of stimulation the histological properties of wounds in. *Wound Repair Regen*.

[20] Cassettari LL (2010). *Efeitos da corrente elétrica contínua isolada e da iontoforese transdermal, associada ao zinco, sobre a resistência mecânica e o conteúdo de hidroxiprolina de cicatrizes realizadas na pele de ratos diabéticos aloxânicos [Mestrado em Agressão, Reparação, Regeneração e Transplantes de Tecidos e Órgãos]*.

[21] Komesu MC, Tanga MB, Buttros KR, Nakao C (2004). Effects of acute diabetes on rat cutaneous wound healing. *Pathophysiology*.

[22] Barbul A, Regan MC (1995). Immune involvement in wound healing. *Otolaryngologic Clinics of North America*.

[23] Mutsaers SE, Bishop JE, McGrouther G, Laurent GJ (1997). Mechanisms of tissue repair: from wound healing to fibrosis. *International Journal of Biochemistry and Cell Biology*.

[24] Mustoe TA, Pierce GF, Thomason A, Gramates P, Sporn MB, Deuel TF (1987). Accelerated healing of incisional wounds in rats induced by transforming growth factor-*β*. *Science*.

[25] Falanga V (1993). Growth factors and wound healing. *Dermatologic Clinics*.

[26] Delamaire M, Maugendre D, Moreno M, Le Goff MC, Allannic H, Genetet B (1997). Impaired leucocyte functions in diabetic patients. *Diabetic Medicine*.

[27] Blakytny R, Jude E (2006). The molecular biology of chronic wounds and delayed healing in diabetes. *Diabetic Medicine*.

[28] Clark RAF (1985). Cutaneous tissue repair: basic biologic considerations. I. *Journal of the American Academy of Dermatology*.

[29] Ortolan EVP, Spadella CT, Caramori C, Machado JLM, Gregorio EA, Rabello K (2008). Microscopic, morphometric and ultrastructural analysis of anastomotic healing in the intestine of normal and diabetic rats. *Experimental and Clinical Endocrinology and Diabetes*.

[30] Rizk NN (1983). Scanning electron microscopy of the structural reconstruction of the abdominal wall after experimental paramedian incision. *Journal of Surgical Research*.

[31] Parizotto NA (1998). *Acão do laser Hélio-Neônio sobre o processo de raparo tecidual: Um estudo do colágeno por microscopia eletrônica de varredura, microscopia de força atômica e espectroscopia por infravermelho [Doutorado em Engenharia Elétrica]*.

[32] Beheregaray WK, Gianotti GC, Leal JS, Garcez T, Contesini EA (2014). Electrical stimulation in experimental wound healing in rabbits. *Ciência Rural*.

[33] Priya B, Rashmi T, Bozena M (2006). Transdermal iontophoresis. *Expert Opinion on Drug Delivery*.

[34] Onodera H, Ikeuchi D, Nagayama S, Imamura M (2004). Weakness of anastomotic site in diabetic rats is caused by changes in the integrity of newly formed collagen. *Digestive Surgery*.

[35] Agren MS, Soderberg TA, Reuterving C-O, Hallmans G, Tengrup I (1991). Effect of topical zinc oxide on bacterial growth and inflammation in full-thickness skin wounds in normal and diabetic rats. *Acta Chirurgica—European Journal of Surgery*.

[36] Agren MS, Chvapil M, Franzén L (1991). Enhancement of re-epithelialization with topical zinc oxide in porcine partial-thickness wounds. *Journal of Surgical Research*.

[37] Agren MS (1990). Studies on zinc in wound healing. *Acta Dermato-Venereologica Supplement*.

[38] Norman JN, Assadulah R, Smith G (1975). Effect of supplements of zinc salts on the healing of granulating wounds in the rat and guinea pig. *Journal of Nutrition*.

[39] Cornwall MW (1981). Zinc iontophoresis to treat ischemic skin ulcers. *Physical Therapy*.

[40] Esen F, Güleç S, Esen H (2006). Exogenous zinc improves blood fluidity but has no effect on the mechanisms of vascular response to acetylcholine iontophoresis in humans. *Biological Trace Element Research*.

[41] Cassettari LL, Dias PCR, Lucchesi AN, de Arruda MF, Spadella CT (2013). Zinc sulphate administered by transdermal iontophoresis improves breaking strength of surgical wounds in skin of alloxan-induced diabetic rats. *Acta Cirurgica Brasileira*.

